# GDNF-based therapies, GDNF-producing interneurons, and trophic support of the dopaminergic nigrostriatal pathway. Implications for Parkinson’s disease

**DOI:** 10.3389/fnana.2015.00010

**Published:** 2015-02-13

**Authors:** Xavier d’Anglemont de Tassigny, Alberto Pascual, José López-Barneo

**Affiliations:** ^1^Instituto de Biomedicina de Sevilla (IBiS), Hospital Universitario Virgen del Rocío/CSIC/Universidad de SevillaSeville, Spain; ^2^Departamento de Fisiología Médica y Biofísica, Facultad de Medicina, Universidad de SevillaSeville, Spain; ^3^Centro de Investigación Biomédica en Red sobre Enfermedades Neurodegenerativas (CIBERNED)Madrid, Spain

**Keywords:** GDNF, Parkinson disease, parvalbumin interneurons, neurotrophic factors, mouse models, dopaminergic system, nigrostriatal pathway, striatum

## Abstract

The glial cell line-derived neurotrophic factor (GDNF) is a well-established trophic agent for dopaminergic (DA) neurons *in vitro* and *in vivo*. GDNF is necessary for maintenance of neuronal morphological and neurochemical phenotype and protects DA neurons from toxic damage. Numerous studies on animal models of Parkinson’s disease (PD) have reported beneficial effects of GDNF on nigrostriatal DA neuron survival. However, translation of these observations to the clinical setting has been hampered so far by side effects associated with the chronic continuous intra-striatal infusion of recombinant GDNF. In addition, double blind and placebo-controlled clinical trials have not reported any clinically relevant effect of GDNF on PD patients. In the past few years, experiments with conditional *Gdnf* knockout mice have suggested that GDNF is necessary for maintenance of DA neurons in adulthood. In parallel, new methodologies for exogenous GDNF delivery have been developed. Recently, it has been shown that a small population of scattered, electrically interconnected, parvalbumin positive (PV+) GABAergic interneurons is responsible for most of the GDNF produced in the rodent striatum. In addition, cholinergic striatal interneurons appear to be also involved in the modulation of striatal GDNF. In this review, we summarize current knowledge on brain GDNF delivery, homeostasis, and its effects on nigrostriatal DA neurons. Special attention is paid to the therapeutic potential of endogenous GDNF stimulation in PD.

## Introduction

Parkinson’s disease (PD) is a progressive, mainly idiopathic and age-related, neuronal disorder that affects as much as 1% of the population over 60 years (de Lau and Breteler, 2006). PD causes severe postural, motor, and physiological impairments that can reduce life expectancy. Although PD is a systemic disease, affecting central and peripheral neurons, the most disabling motor symptoms are due to the progressive death of dopaminergic (DA) neurons in the substantia nigra pars compacta (SNpc), a mesencephalic nucleus that sends projections to the striatum (caudate nucleus (Cd) and putamen) and is involved in motor control. Although pharmacological (pro-DA drugs) and surgical (deep brain stimulation) therapies exist to alleviate PD symptoms (see Tarazi et al., 2014), to date there is no cure for PD despite intense efforts made to develop new protocols, particularly cell replacement therapy, to substitute or protect nigrostriatal cells affected by the disease. The discovery by Lin et al. (1993) of a specific DA neurotrophic factor secreted by rat glial cells -the glial cell line-derived neurotrophic factor (GDNF)- opened a new perspective for PD pathogenesis and therapy. This review will discuss the pros and cons of using GDNF as a treatment for PD, highlighting the potential therapeutic applicability of endogenous brain GDNF activation.

## GDNF administration for treatment of Parkinson’s disease: early observations and clinical trials

GDNF and its structurally related trophic proteins artemin, neurturin and persephin, are distant member of the transforming growth factor-β superfamily (Airaksinen and Saarma, 2002). A wealth of papers based on rodent and non-human primate models have described the benefits of GDNF treatment on nigrostriatal neurons. In early studies, GDNF showed a specific action on survival of rat E16 midbrain DA neurons in culture and proved to be a potent and selective stimulator of dopamine uptake and neurite outgrowth in tyrosine hydroxylase positive (TH+) neurons (Lin et al., 1993). These initial *in vitro* observations led to immediate testing of GDNF effects on PD animal models based on toxin-induced destruction of midbrain DA neurons. Hoffer et al. (1994) used rats unilaterally injected with 6-hydroxydopamine (6-OHDA) in the nigrostriatal pathway. This procedure elicits a rapid and permanent ipsilateral destruction of DA neurons that is manifested by a contralateral rotation pattern in response to low doses of amphetamines, thus accurately reflecting the degree of DA neuronal loss. In 6-OHDA-treated animals, intranigral injection of 100 μg of recombinant human GDNF reduced the rotations by ~4-fold (Hoffer et al., 1994). Similar rescue effects of GDNF were reported in an independent study on the same rat model (Winkler et al., 1996). In 1995, four articles described the potent neurotrophic effects of GDNF on mesencephalic DA (Beck et al., 1995; Tomac et al., 1995a) as well as motor (Oppenheim et al., 1995; Yan et al., 1995) neurons *in vivo*; a year later the first non-human primate data in a 1-methyl-4-phenyl-1,2,3,6-tetrahydropyridine (MPTP)-induced parkinsonian monkey model was published (Gash et al., 1996). GDNF-treated monkeys showed functional improvement of parkinsonian features along with increased levels of striatal dopamine. The benefits claimed by GDNF use were unanimous, although when it came to human patients the initial elation dissipated.

Several human studies have been performed to test the effect of striatal delivery of GDNF through a permanently implanted cannula. The degree of symptomatic relief in these clinical trials has varied from major improvement (Gill et al., 2003; Love et al., 2005; Patel et al., 2005; Slevin et al., 2005) to no benefit at all (Lang et al., 2006). Some patients enrolled in these studies developed neutralizing antibodies as part of an immune response to the recombinant human GDNF treatment (Lang et al., 2006; Tatarewicz et al., 2007), whereas others simply reacted to the placebo in a randomized trial (Lang et al., 2006). In another study, intraventricular GDNF delivery resulted in strong adverse effects (Nutt et al., 2003). A phase II clinical trial, based on improved bilateral intra-putaminal GDNF injection, has recently been launched at the Frenchay Hospital in Bristol (UK) to overcome the inconsistent results previously obtained. Progress to a treatment is hampered by the problem of delivering GDNF to brain cells across the blood-brain barrier (Boado and Pardridge, 2009). Thus, it seems that the simple administration of the GDNF protein does not represent a sustainable treatment for PD and alternative options have to be tested to exploit the benefit of the potent trophic action of GDNF on DA neurons.

## Alternative GDNF-based therapies

**Figure 1:**
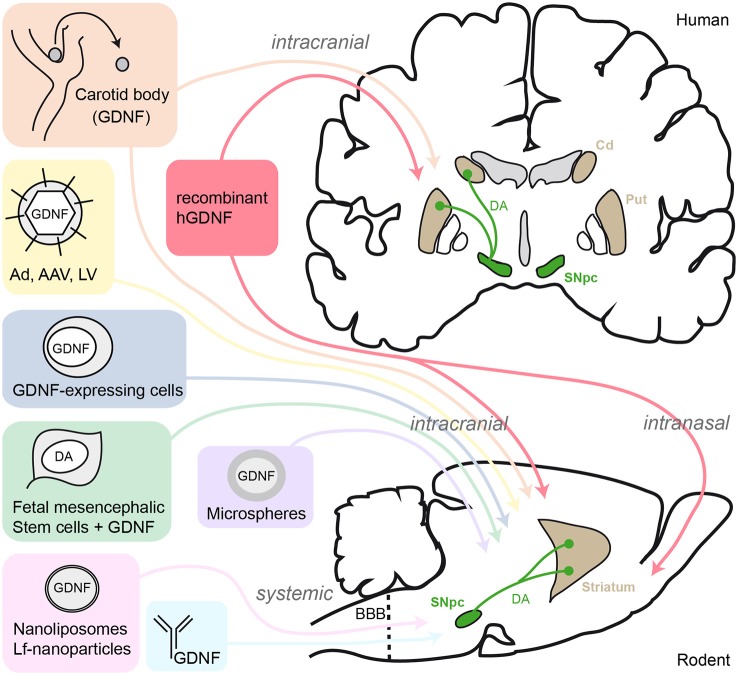
**Schematic summary of GDNF-delivery strategies tested in human PD patients and rodent models**. AAV, adeno-associated virus; Ad, adenovirus; LV, lentivirus; Lf, lactoferrin; SNpc, substantia nigra pars compacta; DA, dopamine; Cd, caudate nucleus; Put, putamen; BBB, blood-brain-barrier.

Overcoming the blood-brain-barrier (BBB) obstacle for GDNF delivery to the brain using a systemic route has became a major technological objective (see Figure 1). Trojan horse approaches were tested by the mean of systemic administration of nanoliposomes engulfing a GDNF plasmid and engineered to cross the BBB via trancytosis after coupling to the transferrin receptor. This resulted to a near complete rescue of the nigrostriatal system from 6-OHDA neurotoxicity in the rat brain (Xia et al., 2008; Zhang and Pardridge, 2009). An attempt to fuse GDNF to a monoclonal immunoglobulin (GDNF-IgG) directed against the BBB cellular component proved to be potent in mice (Fu et al., 2010), but this method failed when it was tested on monkeys as no behavioral improvements were observed (Ohshima-Hosoyama et al., 2012). Biodegradable GDNF-loaded microspheres implanted in the striatum are an interesting alternative to overcome the BBB problem since they sustainably release recombinant GDNF for at least 8 weeks (Jollivet et al., 2004a; Garbayo et al., 2009; Herrán et al., 2013), with long protective effects lasting up to 24 weeks (Jollivet et al., 2004b). Finally, the administration of GDNF by nasal route, using cationic liposomes to increase their residence time through electrostatic interactions at the olfactory epithelium, has recently been tested. Intranasal GDNF given to rats, immediately prior to 6-OHDA lesion, provided significant protection of striatal DA neurons (Migliore et al., 2014).

In parallel with the studies based on the delivery of GDNF peptide, considerable efforts have been made towards the development of *in vivo* gene transfer by recombinant viral vectors expressing the *Gdnf* gene (Figure 1). Bilateral intranigral delivery of adenoviral vector constructs carrying the GDNF sequence (Ad-*Gdnf*) to adult rats prior to 6-OHDA lesion protected DA neurons from toxin-induced cell death (Choi-Lundberg et al., 1997). Although the experimental design was criticized (Pallini et al., 1997), this landmark attempt was encouraging and thus followed by several other studies based on viral vector-driven GDNF strategy in rodent and monkey models (reviewed by Kordower and Bjorklund, 2013). A key study reported that adeno-associated virus (AAV)-*Gdnf* promoted motor recovery of parkinsonian rats when injected in the striatum rather than in the SN region (Kirik et al., 2000). Furthermore, intranigral AAV-*Gdnf* exhibited histological neuroprotection on DA neuronal bodies but DA fibers sprouting and functional recovery occurred only when AAV-*Gdnf* was transduced in the striatum (Kirik et al., 2000, 2004). Several viral vector based strategies have been developed to optimize GDNF production, in particular inducible vectors in order to control the timely expression of GDNF. For instance, injection of a synthetic steroid mifepristone lead to highly increased levels of GDNF expression from the inducible AAV-*Gdnf*. This allowed the recovery of motor function in 6-OHDA lesioned rats, and was associated to DA neuron protection in the SN (Tereshchenko et al., 2014). Another newly reported approach used lentivirus (LV) vectors transgenes fused with a destabilizing domain (DD). The resulting fusion protein is unstable and rapidly cleared by the proteasome unless it is stabilized by trimethoprim (TMP). Thus, peripheral injection of TMP allows DD-GDNF stabilization in the striatum (Tai et al., 2012). When applied to 6-OHDA lesioned rats, the TMP-stabilized DD-GDNF protects the DA nigrostriatal pathway and associated functional behavior (Quintino et al., 2013). Pharmacological modulation of GDNF-expressing viral vectors, still in initial stage of development, is particularly attractive when considering new therapeutic approaches in early disease stages to protect nigrostriatal degeneration and concomitantly prevent adverse effects from sustained high GDNF delivery. Biodegradable nanoparticles encompassing a plasmid DNA coding for GDNF can get through the plasma membrane of neurotensin receptor-expressing cells, such as DA neurons. This non-viral targeted transfection has proved to be efficient when used in rat PD models (Gonzalez-Barrios et al., 2006). A set of experiments combining non-viral gene delivery with systemic route of administration gave promising results. Multiple intravenous injections of a lactoferrin (Lf)-modified vector, expressing human GDNF, protected DA neurons and highly reduced the amphetamine-induced rotational behavior that normally occurs after lesion by 6-OHDA intrastriatal injection (Huang et al., 2009).

Evidently, not all studies have systematically reported positive effect of viral GDNF vectors. Indeed, an herpes simplex virus (HSV)-derived vector overexpressing GDNF presented toxic effects while masking the potential benefits of GDNF (Monville et al., 2004). Intranigral lentiviral injection of a vector expressing the A30P mutant human α-synuclein provoked a selective and progressive degeneration of the nigrostriatal DA neurons in the treated rats (Lo Bianco et al., 2002). Preventive treatment by LV-GDNF vector, successfully used in a monkey PD model (Palfi et al., 2002), failed to modulate nigrostriatal degeneration induced by the α-synuclein toxicity (Lo Bianco et al., 2004). Surprisingly, the use of a tetracyclin-dependent LV-GDNF expression in the striatum in normal rats provoked a dramatic down-regulation of TH protein expression (Georgievska et al., 2004).

Cell-based GDNF therapy, i.e., transplantation of GDNF-expressing cells, has also been extensively tested. Two main strategies have been used so far: (i) introduction of GDNF-secreting cells in the lesioned nigrostriatal system; and (ii) transplantation of DA-producing cells in association with GDNF treatment to protect and to increase survival of grafted cells. Successful intrastriatal transplantation of primary astrocytes engineered to express GDNF prevented 6-OHDA-induced DA neuronal death (Cunningham and Su, 2002). Interestingly, low levels of GDNF released by these astrocytes (~5 pg/g of striatum) provided a remarkable robust neuroprotection. Neural stem cells engineered to synthesize GDNF were also successfully used to limit DA neuron degeneration in a 6-OHDA lesion mouse model (Åkerud et al., 2001). Encapsulated GDNF-producing cells may represent a valuable option since they will not migrate out of the targeted region, the caudate-putamen, and can still be removed in the event that some adverse effects may occur (reviewed by Lindvall and Wahlberg, 2008). Pioneer work from Tseng et al. (1997) used polymer-encapsulated fibroblasts engineered to overexpress GDNF prior to be transplanted next to the SN. Nanogram levels of continuous GDNF release completely prevented degeneration of DA neurons induced by medial forebrain bundle axotomy. Alternatively, trophic factors-producing tissues, such as the carotid body (CB), have been used as a source of GDNF. The CB is highly DA, bilateral, O_2_-sensing organ that contains cells which produce unusual high levels of GDNF (López-Barneo et al., 1999; Villadiego et al., 2005). Intrastriatal transplantation of CB cells produces clear neuroprotective effects on DA neurons in rodent parkinsonian models (Espejo et al., 1998; Muñoz-Manchado et al., 2013), and amelioration, with indications of biological effects, in PD patients (Mínguez-Castellanos et al., 2007). However, the therapeutic action of CB is limited by the small amount of tissue available. To overcome this limitation, new stem cell-based procedures are being assayed to expand CB tissue before transplatation (see Pardal et al., 2007; Platero-Luengo et al., 2014). The combination of GDNF delivery and fetal DA grafts, to improve survival of transplanted cells, has been largely tested in animal models (Rodriguez-Pallares et al., 2012; Kauhausen et al., 2013), as well as in PD patients (Mendez et al., 2000). GDNF promoted survival of fetal mesencephalic cell transplants in the striatum of 6-OHDA-lesioned rats, which was associated with functional improvement (Yurek et al., 2009). However, this beneficial effect was limited in time, as 6 months later the association of grafted cells/LV-GDNF failed to support DA neuron survival. Moreover, LV-GDNF induced some down regulation of TH in the grafted cells. In similar experimental conditions, GDNF had no effect on fetal mesencephalic graft outgrowth when compared to other growth factors such as bFGF (Törnqvist et al., 2000). GDNF has also been used to increase DA differentiation and survival of embryonic (Buytaert-Hoefen et al., 2004) or bone marrow stromal (Dezawa et al., 2004) stem cell-derived DA neurons prior to transplantation. This procedure, that represents an indirect use of GDNF, substantially alleviated the rotation behavior induced by amphetamines in 6-OH dopamine-lesioned rats. However, the use of GDNF to drive stem cell-derived neuronal cells to produce DA is a procedure that calls for caution, as safety of progenitor cell transplants is always a key concern. Optimization of DA neuron maintenance and GDNF delivery protocols has permitted recent preclinical advances in the field. DA cells from ventral mesencephalon of young donors (embryonic day 10) transplanted homotopically in the nigral region, combined with the intrastriatal injection of a AAV-GDNF, allowed graft survival, integration into the medial forebrain bundle circuitry to innervate the striatum, and functional motor recovery (Kauhausen et al., 2013). Together, the data summarized in this section support a beneficial neuroprotective action of exogenous GDNF on DA nigrostriatal neurons.

## GDNF signaling on dopaminergic neurons

**Figure 2:**
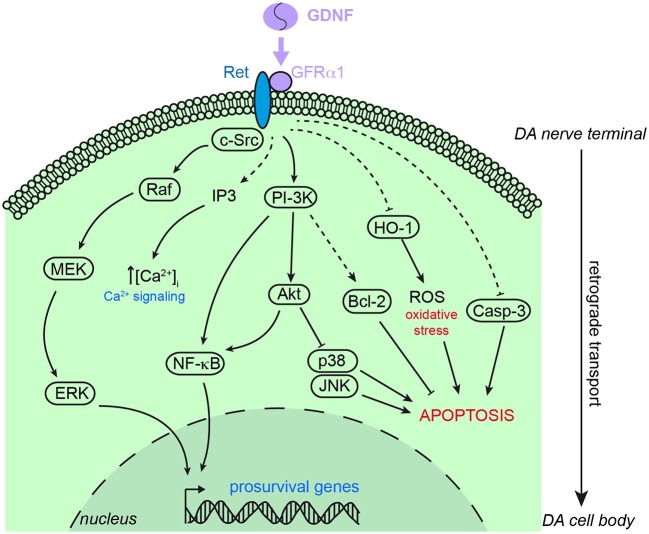
**Schematic representation of the main signaling pathways involved in the neuroprotective action of GDNF on dopaminergic neurons**. GDNF principally stimulates the binding of GFRα1 and Ret to trigger intracellular signaling cascades leading to pro-survival genes expression, calcium signaling and pro-apoptosis factors inhibition. Akt, protein kinase B; Bcl-2, B cell lymphoma 2; Casp-3, caspase 3; c-Src, proto-oncogene tyrosine-protein kinase Src; ERK, extracellular signal-regulated kinase; HO1, heme oxygenase 1; IP3, inositol tris-phosphate; JNK, c-Jun N-terminal kinase; MEK, mitogen extracellular signal-regulated kinase; NF-κB, nuclear factor kappa B; PI-3K, phosphatidylinositol 3 kinase; Raf, Raf kinase; ROS, reactive oxygen species. Dashed arrows indicate indirect stimulation or inhibition.

GDNF shares the receptor tyrosine kinase rearranged during transcription (Ret) with artemin, neurturin and persephin. Ret activation requires association to a second glycosylphosphatidyl inositol-anchored protein named GDNF family receptor α (GFRα), of which four subtypes have been identified with different affinities for ligands of the GDNF-family. The GDNF homodimer specifically binds to two GFRα1 to form a high affinity complex with the recruitment of Ret proteins (Bespalov and Saarma, 2007). GDNF displays lower affinity for GFRα2 and GFRα3. The Ret-GFRα1 complex formation induces transphosphorylation of Ret tyrosine kinase residues which, in turn, activates downstream signaling molecules (Figure 2) such as the mitogen-activated protein kinase (MAPK) and the phosphatidylinositol 3-kinase (PI3K)/Akt (Airaksinen and Saarma, 2002). *In vitro* studies suggest that the protective effect of GDNF on DA neurons involves the activation of the MAPK and PI3K intracellular pathways (Ugarte et al., 2003; Onyango et al., 2005). Aging mice (26 months) carrying a partial deletion of *Gfrα1* (heterozygous), show a decrease in TH fiber density in the striatum accompanied by a lower number of TH+ neurons in the SN. Additionally, these mice exhibit increased sensitivity of nigrostriatal DA neurons to MPTP toxicity (Boger et al., 2008). These observations suggest a pivotal role of GFRα1 in the trophic protection by GDNF signaling. Specific ablation of Ret in DA neurons (using a dopamine transporter-Cre/Ret-flox mice) results in progressive loss of nigrostriatal DA neurons. Spontaneous decrease of TH+ cells in the SNpc and striatal innervation occurred in these mice and this was associated with increased number of activated glial cells, a sign of CNS injury (Kramer et al., 2007). GDNF signaling also utilizes c-Src kinase to promote neurites outgrowth (Encinas et al., 2001). Although the GFRα1/Ret complex is the most studied GDNF receptor, it is known that this trophic factor can also bind to alternative signaling system, e.g., NCAM (Paratcha et al., 2003). This would explain why ablation of *Ret* does not produce a phenotype similar to GDNF-deficiency (see Pascual et al., 2011).

The data summarized in the previous paragraph strongly suggest the requirement of direct GDNF trophic signaling to the DA neurons for their survival. Ret and GFRα1 mRNA expressions are up-regulated in the SNpc shortly after 6-OHDA lesion, a trophic response to drug toxicity. After 3 to 6 days, the level of expression of both Ret and GFRα1 mRNA decreased dramatically, which could be explained by the loss of DA neurons observed after 6 days in this rat PD model (Marco et al., 2002). Ret is not specific to GDNF but its activation is also enhanced by other ligands such as GM1 ganglioside (Newburn et al., 2014). This observation denotes a possible pharmacological induction of the GDNF signaling cascade to promote a trophic response. The canonical neurotrophic factor action requires retrograde communication from the axon terminals to neuron cell bodies, partly explained by the “signaling endosome hypothesis” where the activated receptor is internalized and transported via the microtubules machinery for cytosolic and nuclear signaling (Howe and Mobley, 2005; Ibáñez, 2007). GDNF is no exception to this rule as it has been demonstrated that ^125^I-GDNF injected into the rat striatum is retrogradely transported to the cell body of SNpc neurons (Tomac et al., 1995b).

The use of *Gdnf*-null mice has provided valuable data regarding the role of endogenous GDNF on DA neuron survival. Mice carrying GDNF deletion do not survive after birth due to lack of the entire enteric nervous system and kidney agenesis (Moore et al., 1996; Pichel et al., 1996; Sánchez et al., 1996). However, embryonic development of the midbrain DA nigrostriatal pathway is not affected by the lack of GDNF (Sánchez et al., 1996). Mice with partial deletion of *Gdnf* (*Gdnf*^+/−^) suffer from higher neuro-inflammation and loss of TH-positive neurons with aging (Boger et al., 2006) or following lipopolysaccharide (LPS) treatment (Granholm et al., 2011). However, whether GDNF might serve as an important target-derived neurotrophic factor for adult nigral DA neurons has remained unknown until conditional GDNF-KO mice were generated. Inducible CRE-LoxP *Gdnf*-null mice were engineered to bypass the developmental lethality caused by GDNF loss. In this model, a *floxed-Gdnf* allele was deleted in adulthood by tamoxifen-induced Cre recombinase activation, leading to a marked decrease of GDNF expression in the striatum (Pascual et al., 2008). Strikingly, these mice showed a progressive and selective death of the catecholaminergic neuronal population in the substantia nigra (SN), ventral tegmental area, and locus coeruleus with associated locomotor dysfunction (Pascual et al., 2008). These data further support the notion that adult mammalian mesencephalic catecholaminergic neurons rely on the continuous input of endogenous GDNF, an observation that remains to be demonstrated with other animals models and in the human brain.

## Mechanisms involved in the protective effect of GDNF

It is postulated that GDNF protects the DA nigrostriatal system by interacting with several cellular pathways involved in apoptosis, metabolism, and redox homeostasis (see Figure 2). GDNF may prevent apoptosis in the DA neuron population by directly up-regulating the anti-apoptotic proteins B cell lymphoma 2 (Bcl-2) and Bcl-X via PI3K signaling (Sawada et al., 2000). The neuroprotective action by GDNF on the nigrostriatal system might also involve the activation of protein kinase CK2 as demonstrated in parkinsonian rats (Chao et al., 2006). Moreover, GDNF induces nuclear factor κB (NF-κB) pathways to promote neuronal survival from toxic insults (Cao et al., 2008). Other targets of GDNF are caspase-3 and the endoplasmic reticulum stress-related genes. Treatment of primary mesencephalic rat cultures with lactacystin inhibits the ubiquitin-proteasome system and leads to apoptosis of DA neurons. However, pretreatment with GDNF prevents DA neuronal death by suppressing caspase-3 activation and endoplasmic reticulum stress (Li et al., 2007). Intrastriatal infusion of GDNF prevents lactacystin-induced DA neuron loss by inhibiting the pro-apoptotic molecules Jun N-terminal kinase (JNK) and p38 and activating the pro-survival Akt and MAPK pathways (Du et al., 2008).

As it occurs in the classical neurotrophic models, GDNF promotes the DA phenotype in DA neurons, and in this way exerts some of its neuroprotective actions. GDNF seems to increase cellular levels of transcription factors, such as Nurr1 and Pitx3, involved in the expression of a set of genes—TH, vesicular monoamine transporter (*Vmat2*), dopamine transporter (*Dat*) and aromatic L-amino acid decarboxylase (*Aadc*)—involved in dopamine metabolism, (Lei et al., 2011). When added to the culture medium of midbrain-derived neural stem cells (mdNSCs), GDNF induced a DA phenotype associated with Nurr1 and Pitx3 up-regulation. Transplantation of these cells into the striatum of 6-OHDA-injected rats greatly prevented the amphetamine-induced contralateral rotation in the lesioned animals (Lei et al., 2011).

Although the causes of DA neuron degeneration in PD remain unclear, mitochondrial dysfunction and oxidative stress induced by reactive oxygen species (ROS) are known to have a pathogenic role early in the disease process (Subramaniam and Chesselet, 2013). Interestingly, striatal GDNF administration moderately enhances the activity of certain enzymes involved in the enzymatic detoxification of ROS: superoxide dismutase, catalase and glutathione peroxidase (Chao and Lee, 1999). Moreover, GDNF administration in the rat striatum prevents 6-OHDA-induced ROS formation, evidenced by protein carbonyls and 4-hydroxynonenal, and thus protects DA neurons from oxidative stress (Smith and Cass, 2007). GDNF seems to negatively regulate the expression of heme oxygenase-1 (HO-1) to reduce oxidative stress (Saavedra et al., 2005).

A proteomic analysis revealed 46 specifically regulated proteins in the striatum of MPTP mice 4 and 72 h after striatal GDNF injection. These proteins are related to cell differentiation, system development, cell structure and motility, energy pathways, transport, apoptosis, cell proliferation and response to stress-regulating genes (Hong et al., 2009). However, none of them are involved in GFRα1-Ret downstream-activated pathways. Taking into account that post-transcriptional modification, such as phosphorylation, were not detected with the aforementioned method, a thorough proteomic examination of posttranslational modifications elicited by GDNF on DA neurons would probably provide relevant information for understanding the neuroprotective action of GDNF. It has been reported that striatal GDNF inhibits Shh production by DA neurons and, in turn, Shh released at the striatal DA terminals down-regulates *Gdnf* gene expression (Gonzalez-Reyes et al., 2012). This concept is attractive, as the mutual repression of Shh and GDNF would allow DA neurons to dynamically control neurotrophic factor production in the striatum. The level of striatal *Gdnf* mRNA, and the number of GDNF-expressing parvalbumin-positive (PV+) interneurons (see below) do not seem to be affected by MPTP-derived lesions of nigrostriatal neurons (Hidalgo-Figueroa et al., 2012). However, to what extent the integrity of the DA nigrostriatal pathway modulates the survival and activity of GDNF-producing striatal interneurons is under debate. Although much progress has been done regarding the molecular mechanism of GDNF neurotrophic/neuroprotective action, whether the intracellular pathways involved are the same in normal and lesioned cells and to what extent GDNF production is cell autonomous or depend on the activity of the relevant neuronal networks are fundamental questions yet to be resolved.

In addition to its well-established neurotrophic role, GDNF may also modulate the activity of DA nerve terminals at the basal ganglia. Amperometric recordings from midbrain DA neurons showed that exposure to GDNF increases quantal release of catecholamines as well as the density of axonal varicosities (Pothos et al., 1998). GDNF enhances basal levels and release of DA and DA metabolites evoked by potassium or amphetamine in primary cultured ventral midbrain (VM) DA neurons (Wang et al., 2001), striatal slices (Gomes et al., 2006), and striatal synaptosomes (Gomes et al., 2009). Similar effects of GDNF have also been observed *in vivo* by microdialysis measurements (Hebert et al., 1996; Xu and Dluzen, 2000; Cass and Peters, 2010). Therefore, GDNF may not only prevent DA neurons from degeneration but also potentiate DA release and turnover by some as yet unknown mechanism.

## Endogenous GDNF expression: striatum

Knowledge of where and when GDNF is expressed in the adult brain is fundamental to understand the physiological role of this trophic factor and the mechanisms that regulate its synthesis. Eventually, this could make it possible to pharmacologically stimulate endogenous GDNF production as a way to increase the level of GDNF available at the striatal DA nerve terminals. Unfortunately, studies on GDNF expression performed with antibodies are challenged by specificity considerations. However, there are several studies in which either *Gdnf* mRNA expression was analyzed by *in situ* hybridization (ISH), or mouse models with reporter genes were used to estimate *Gdnf* promoter activity. In rodents, *Gdnf* mRNA is broadly expressed in the developing embryo (Golden et al., 1999), although in adult mice its expression is rather limited to few organs, with the highest content found in the ovary and testis. In the adult rodent brain, *Gdnf* mRNA expression is consistently observed in restricted discrete cells of the striatum, thalamic structures, nucleus accumbens, cerebellum and hippocampus (Schaar et al., 1993; Nosrat et al., 1996; Trupp et al., 1997). Using a β-gal reporting mouse model (Sánchez et al., 1996), GDNF expression in adult mice brain was restricted to the dorsal and ventral striatum, the anteroventral nucleus of the thalamus, the septum and, interestingly, the subcommissural organ (Pascual et al., 2008). Curiously, GFRα1 and Ret do not share the same expression pattern than GDNF and are broadly expressed in the adult CNS. Noteworthy, GDNF receptor mRNAs are not detected in the striatum, but highly expressed in the SNpc (Trupp et al., 1997), which again supports that GDNF may specifically act on SNpc DA neurons that project to the striatum. This also indicates that no other striatal cells could benefit from its trophic action. GDNF protein levels have been measured by enzyme-linked immunosorbant assay (ELISA) in lysates of caudate/putamen, SN, cerebellum, frontal cortex, and the cerebrospinal fluid (CSF) of PD and non-PD postmortem human brains. GDNF concentration in the range of 40–70 pg/mg total protein was relatively constant between control and PD patients in the SN and Cd and putamen, with lower concentrations reported in the cerebellum and the frontal cortex (10–15 pg/mg). However, GDNF was no detected in the CSF (Mogi et al., 2001). Additionally, polymorphisms in the GDNF gene have been found in PD and non PD patients with no apparent correlation between mutation and disease (Wartiovaara et al., 1998). In another study, depletion of GDNF, but no other neurotrophic factors, was detected in the SN of parkinsonian patients (Chauhan et al., 2001). Although these results must be taken with caution as they are based on immunohistochemical analyses, they suggest that down regulation of GDNF might participate in the onset of PD pathophysiology. However, whether alterations in GDNF synthesis and release have any causative pathogenic role in PD is for the moment unknown.

**Figure 3:**
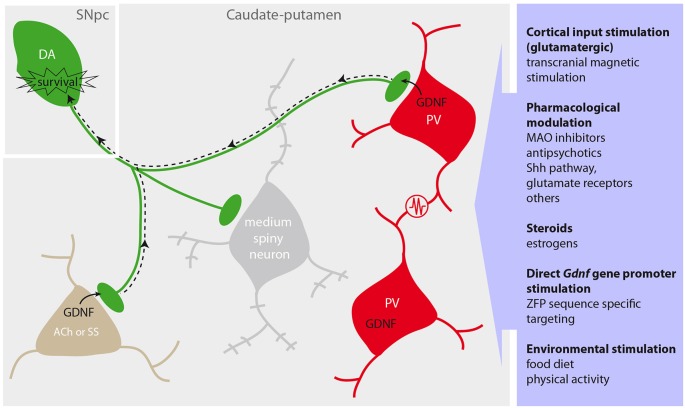
**Protection of the dopaminergic nigrostriatal pathway by striatal GDNF and activation of endogenous GDNF production**. Dopaminergic (DA) neurons (*green*) located in the substantia nigra pars compacta (SNpc) innervate the caudate-putamen to modulate the activity of GABAergic medium spiny neurons (*gray*), parvalbumin (PV)-positive interneurons (red) and other cholinergic (ACh) or somatostatin (SS) interneurons (brown). PV neurons form an ensemble of synchronized cells through multiple dendrodendritic electrical synapses (*resistance* in the scheme), and release GDNF at the nerve terminals to provide trophic support to DA neurons via retrograde signaling (dotted arrow). Proposed strategies to enhance the endogenous GDNF production are summarized in the purple box (right).

There are few studies focused on the cell distribution of striatal GDNF. In an ISH-based study over 60% of the choline acetyl-transferase (ChAT) positive interneurons were reported to express *Gdnf* mRNA (Bizon et al., 1999). In the same study, a significant fraction (17–42%) of GABAergic neurons expressed *Gdnf* mRNA, however it did not discriminate between the medial spiny neurons (the most abundant cells in the striatum) and the GABAergic interneurons. As PV+ interneurons represent only a small fraction of GABA-positive cells, it could be concluded from these data that PV+ neurons account for a small proportion of striatal cells expressing GDNF (Bizon et al., 1999). However, in this study a majority of PV+ cells expressed NGF and acidic fibroblast growth factor (FGF1), which are thought to provide trophic protection to excitotoxic insult. Interestingly, some cells were found highly co-expressing GDNF and FGF1 (Bizon et al., 1999). In contrast with these observations, the use of a *Gdnf-LacZ* mouse model (Sánchez et al., 1996) unveiled a different population of GDNF-expressing cells in the striatum. *Gdnf* promoter-driven *LacZ* expression, revealed by β-galactosidase activity (XGal staining), demonstrates that *Gdnf* is expressed in more than 80% of striatal PV+ GABAergic interneurons. Moreover, ~95% of the GDNF-positive striatal neurons are PV+, while the remaining GDNF+ cells are either cholinergic (ACh) or somatostatinergic (SS) interneurons (Hidalgo-Figueroa et al., 2012; see Figure 3). As yet there is no explanation for the discrepancy between these two studies performed in different models of rat (Bizon et al., 1999) and mouse (Hidalgo-Figueroa et al., 2012). However the particularly scattered distribution of PV+ cells throughout the mouse striatum, their electrical coupling by dendro-dendritic gap junctions (Fukuda, 2009) and their high resistance to excitotoxicity, make them a target of choice for pharmacological modulation. On the other hand, although the number of ACh+ and GDNF+ cells does not seem to be too high, they may have a significant contribution to striatal GDNF homeostasis, as degeneration of cholinergic interneurons following the injection of the cholinotoxin AF64α results in a 30% reduction in striatal GDNF protein content (Gonzalez-Reyes et al., 2012). This decrease of GDNF production might be directly inferred to the loss of ACh+ interneurons, or a consequence of a drop of cholinergic input to the PV+ interneurons (Chang and Kita, 1992). Despite these recent advances in the identification of GDNF-producing interneurons in the rodent striatum, the nature of the cells that produce GDNF in the human striatum remains as yet unidentified.

In the lesioned striatum, reactive astrocytosis occurs in parallel to an increase in GDNF expression (Nakajima et al., 2001). Similarly, in the DA-depleted striatum, reactive astrocytes results in expression of *Gdnf* mRNA, as shown by both quantitative RT-PCR and ISH (Nakagawa and Schwartz, 2004). However, in the *Gdnf-LacZ* mice, none of the GDNF expressing cells are of astrocyte or microglia origin 7 and 21 days post-MPTP, despite a significant increase of the astrocytic population occurred (Hidalgo-Figueroa et al., 2012). Unilateral nigrostriatal lesions with 6-OHDA produce a 50% decrease in the number of PV+ neurons in the ipsilateral side in comparison with the contralateral side (Proschel et al., 2014). This brings an interesting contradiction with the MPTP-treated *Gdnf-LacZ* mice that displayed no difference in PV/GDNF expression in the injured striatum (Hidalgo-Figueroa et al., 2012). These differences may be due to the use of rats vs. mice and distinct parkinsonian models (neurotoxic drugs and route of administration).

## Stimulation of striatal endogenous GDNF production

Since GDNF has a potent neurotrophic effect on DA neurons and it is highly expressed in the striatum, pharmacological or physical interventions aiming at up-regulating endogenous GDNF production are of major potential medical relevance. Several drugs have been tested to boost striatal GDNF expression thus far (see Table 1). For instance, a weeklong systemic injection of 1,25-dihydroxyvitamin D3 (calcitriol) induced *Gdnf* mRNA and protein expression in the rat striatum, presumably via the activation of vitamin D receptors. Longer treatment with calcitriol prevented DA neuron loss in 6-OHDA-lesionned rats (Smith et al., 2006). Monoamine oxidase (MAO) inhibitors rasagiline and selegiline, broadly used to treat PD patients, up-regulate *in vitro* GDNF expression via NF-κB internalization (Mizuta et al., 2000; Maruyama et al., 2004; Bar-Am et al., 2005). It would be interesting to test these MAO inhibitors *in vivo*. Valproic acid, an anti-epileptic drug, induces GDNF secretion in the culture medium of rat astrocytes, which partially prevents DA cell loss after LPS or MPTP treatment (Chen et al., 2006). Valproate is a powerful histone deacetylase inhibitor, therefore facilitating chromatin relaxation and transcriptional activation, which is suggested to facilitate transcription of neurotrophic factors (Harrison and Dexter, 2013). Indeed, treatment with histone deacetylase inhibitors increased *Gdnf* and *Bdnf* expression and preserved DA neuronal function from MPTP injury. Moreover, valproate induced a marked increase in *Gdnf* promoter activity and promoter-associated histone H3 acetylation (Wu et al., 2008). Other mood stabilizer drugs have been reported to trigger GDNF release by rat glioblastoma cell line (see Table 1 for details). In any case, these data must be interpreted cautiously, as rat cortical primary astrocyte and cell line cultures used in these studies are experimental models very different from the striatum *in situ*.

**Table 1:** ***In vivo* and *in vitro* pharmacological tests employed to modulate the endogenous GDNF production**.

Drug or stimulus	Administration	Model	Areas (or origin)	Duration	GDNF detection	GDNF levels	Reference
**MAO inhibitors**
Rasagiline	Culture medium	SH-SY5Y cells		3–24 h	ELISA, WB, Q RT-PCR	> 10 fold ↑	Maruyama et al. (2004), Bar-Am et al. (2005)
Selegiline	Culture medium	Mouse astrocytes		24 h	ELISA	≈ 10 fold ↑	Mizuta et al. (2000)
(-)-deprenyl	Intrastriatal	MPTP mouse	St	30 min	RT-PCR	≈ 2 fold ↑	Tang et al. (1998)
**Antidepressants, antipsychotics**
Valproate	Culture medium	Rat astrocytes	(VM)	24–48 h	ELISA, Q RT-PCR	265% ↑	Chen et al. (2006)
Amitriptyline, fluoxetine	Culture medium	Rat C6 glioblastoma cells		48 h	ELISA, RT-PCR	> 10 fold ↑	Hisaoka et al. (2001)
Serotonine	Culture medium	Rat C6 glioblastoma cells		48 h	ELISA	≈ 5 fold ↑	Hisaoka et al. (2004)
Quetiapine, clozapine, haloperidol	Culture medium	Rat C6 glioblastoma cells		24–48 h	ELISA	up to 4 fold ↑	Shao et al. (2006)
**Chinese medicinal plants-derived molecules**
Echinacoside	i.g.	MPTP mouse	VM	14 days	WB	≈ 2 fold ↑	Zhao et al. (2010)
Puerarin	i.p.	6-OHDA rat, MPTP mouse	St	10 days	IHC, ELISA	≈ 1.5 fold ↑	Zhu et al. (2010, 2014)
Naringin	i.p.	MPTP rat	SN	7 days	WB, IHC	≈ 1.5 fold ↑	Leem et al. (2014)
**Miscellaneous**
Ibogaine, Noribogaine	i.p. i.c.	Rat, mouse	VM	1–24 h	ELISA	≈ 3 fold ↑	He et al. (2005)
	Culture medium	SH-SY5Y cells		1–12 h	ELISA, RT-PCR	> 10 fold ↑	He et al. (2005), Carnicella et al. (2010)
Glutamate receptor 3 agonist (LY379268)	i.p.	Mouse	St, Ctx	6 h	ISH, Q RT-PCR, WB	up to 3 fold ↑	Battaglia et al. (2009)
	Culture medium	Mouse astrocytes	(St), (Ctx)	24 h	WB	≈ 1.7 fold ↑	Battaglia et al. (2009)
AS101	i.c.	6-OHDA Rat	SN	72 h	RT-PCR	≈ 2 fold ↑	Sredni et al. (2007)
1,25-dihydroxyvitamin D3 (Calcitriol)	i.p. s.c.	6-OHDA Rat	St, SN	8 days	ELISA	37%↑ (SN)—(St)	Smith et al. (2006)
hGDNF-ZFP	intrastriatal	6-OHDA Rat	St	4 weeks	Affymetrix, ELISA	up to 4 fold ↑	Laganiere et al. (2010)
PRE-084	s.c.	6-OHDA Mouse	St, SN	7–35 days	WB	37%↑ (SN) 14%↑ (St)	Francardo et al. (2014)
17-β-estradiol	s.c. osmotic pump	6-OHDA Rat	St, SN	8–10 days	WB	≈ 1.5 fold ↑	Campos et al. (2012)

*VM, ventral midbrain; SN, substantia nigra; St, striatum; i.p., intraperitoneal; i.c., intracranial; i.g., intragastric; s.c. subcutaneous; Ctx, cortex; ELISA, enzyme-linked immunosorbant assay; WB, western blot; Q RT-PCR, quantitative reverse transcription polymerase chain reaction; ISH, *in situ* hybridization; IHC, immunohistochemistry*.

Noribogaine, a metabolite of the naturally occurring alkaloid ibogaine, bears anti-addictive effects on alcohol and other drugs consumption. In rats, the effect of ibogaine on the reduction of ethanol intake is located in the ventral tegmental area a DA mesencephalic region medial to the SN. Systemic injection of ibogaine stimulates *Gdnf* mRNA expression in the midbrain of both rats and mice, and when added to the SH-SY5Y adrenergic cell line (He et al., 2005; Carnicella et al., 2010). Although ibogaine/noribogaine is known to act as an agonist to 5-HT2A and κ-opioid receptors and as an antagonist to NMDA receptors, the mechanism by which it induces *Gdnf* mRNA expression remains to be deciphered. Another potential stimulant of *Gdnf* mRNA and protein expression in mouse striatal neurons is the metabotropic glutamate receptor 3 agonist LY379268 (Battaglia et al., 2009). The organotellurium compound AS101 exerts diverse biologic activities and holds great potential in PD. Systemic application of this immunomodulator prevents neurotoxicity and behavioral deficits induced by 6-OHDA striatal injections in rats. Besides activation of the Ras-Raf-MEK-Erk cascade leading to cell growth and survival, AS101 up-regulates GDNF levels by inhibiting interleukin-10 in primary astrocyte cultures as well as in the rat SN (Sredni et al., 2007). It is surprising, however, that this compound has not been further studied in regard to its potential effect on GDNF expression.

Chinese medicinal plants also bring interesting molecules such as echinacoside, a polyphenol natural product that when injected peripherally alleviates MPTP-induced DA neuronal loss. Echinacoside stimulates GDNF and BDNF and prevents MPTP-induced apoptosis (Zhao et al., 2010). Puerarin, from the roots of a kudzu plant *Pueraria lobata*, partially prevents the chemically-induced DA neurodegeneration in mice and rats, and stimulates striatal GDNF (Zhu et al., 2010, 2014). Naringin is another recent example of a plant pigment (flavonoid) present in grapefruits that seems to stimulate GDNF in the SN of MPTP-treated mice (Jung et al., 2014; Leem et al., 2014).

An elegant strategy used to activate endogenous GDNF is based on an engineered zinc-finger protein (ZFP) that specifically activates the GDNF promoter (Laganiere et al., 2010). In this work, a six ZFPs sequence carried by an AAV vector was designed to target rat, human and monkey *Gdnf* promoters (hGDNF-ZFP). Microarray data from *in vitro* assays showed a very specific increase of *Gdnf* mRNA expression while the rest of the genomic activity remained unchanged. hGDNF-ZFP infused into the striatum of normal adult rats 4 weeks before triggering neurotoxicity by a 6-OHDA striatal injection, increased GDNF production in the striatum and improved motor activity in lesioned rats (Laganiere et al., 2010). This methodology could be potentially applicable to prevent DA neuron degeneration in genetic cases in which the disease can be diagnosed before appearance of the clinical symptoms. Whether hGDNF-ZFP induces GDNF expression in the striatal cells that normally synthetize the trophic factor, or if other cell types are also put to contribution, is a point that needs to be clarified. Recently, stimulation of the intracellular Sigma-1 receptor (Sig-1R) by the agonist PRE-084 (Su et al., 1991) showed neurorestorative properties in 6-OHDA-treated mice (Francardo et al., 2014). PRE-084 also induced a moderate, but significant, increase of GDNF protein in the striatum (~6% over vehicle treatment) and in the SN (~14%) whereas no difference was observed in the Sig-1R-*null* mice (Francardo et al., 2014). Quantification with inadequately characterized anti-GDNF antibodies remains a weak point in several of these studies (Battaglia et al., 2009; Di Liberto et al., 2011; Campos et al., 2012; Lee et al., 2013; Francardo et al., 2014). Such antibodies need to be tested on GDNF-KO tissue extracts as they may give false positive bands of the expected molecular size (authors’ unpublished observation), and this may contribute to overstatement on the efficiency of certain drugs in stimulating GDNF expression.

In parallel to the pharmacological agents, noninvasive approaches are also being considered to stimulate endogenous brain GDNF production. *In vitro* analysis has revealed that GDNF is secreted both tonically and after depolarization of cells with high K^+^, suggesting that *in vivo* GDNF could be released in an activity dependent manner (Lonka-Nevalaita et al., 2010). Transcranial magnetic stimulation (TMS) has been used for some time with little insights regarding its actual effect on neurons. A recent study made an attempt to use TMS on rats to assess the effect on GDNF production. Repeated TMS (rTMS), at 10 Hz, during 20 min for 4 weeks proved to be beneficial to unilaterally 6-OHDA-lesioned rats with improvement of behavioral test scores, increase of SNpc TH+ neuron number and fiber density as well as GDNF, NGF and PDGF levels in the striatum (Lee et al., 2013). However, the mechanisms leading to the positive action of rTMS on striatal neurotrophin expression and the associated neurorestorative effect are unknown. Electroconvulsive shock (ECS), a standard psychiatric therapy provoking seizures to provide relief from psychiatric illnesses, is known to improve motor function in PD animal models. ECS prevents neurodegeneration of the DA nigrostriatal pathway observed after 6-OHDA injections. Daily ECS treatment to healthy rats for 7 days stimulates GDNF protein expression in the SN but not in the striatum (Anastasia et al., 2007). Moreover, anti-GDNF IgG inhibits the neuroprotective effect of chronic ECS treatment (Anastasía et al., 2011). It is however not clear how GDNF is up-regulated in the SN since its expression is located in the striatum where no change in protein expression is observed after ECS. A far-fetched explanation would involve the participation of a large ECS-induced glutamate release, which may stimulate GDNF expression and release by the surrounding astrocytes (Yamagata et al., 2002).

Finally, physical exercise (Zigmond et al., 2009), and food restriction diets (Maswood et al., 2004), have both been suggested to have a neuroprotective effect. For example in rats, placing a cast to immobilize the limb ipsilateral to the 6-OHDA injection, thus forcing the use of the contralateral limb, reduces behavioral deficits and DA neuron loss in the lesioned striatum. This also increases GDNF protein content in the striatum (Cohen et al., 2003). Protective effect of exercise on the nigrostriatal DA system associated to an increase of GDNF protein in the 6-OHDA lesioned striatum has been reported in other studies (Tajiri et al., 2010; Lau et al., 2011). Yet, it remains unexplained how exercise can positively modulate GDNF expression, as well as other growth factors, in the striatum and SN. Altogether, the data summarized in this section demonstrate that activation of endogenous GDNF is feasible and therefore further research should be done to determine what methodology, or combination of techniques, can produce more consistent protection for DA neurons and terminals (Figure 1).

## Concluding remarks

Two decades have passed since the discovery of GDNF and much advance has been produced regarding its cellular effects and neuroprotective action on DA neurons. However, it still remains unclear which are the main factors determining GDNF production by brain cells and whether GDNF can effectively be used as a therapeutic agent for PD. Despite intense preclinical research and some clinical studies have been performed, intrastriatal delivery or systemic administration of GDNF have failed so far to provide robust and reproducible methodologies applicable to a large number of PD patients. Intrastriatal transplantation of GDNF-producing cells has worked well in animal models but is still confronted with several limitations (e.g., graft stability, cell survival, and sufficient cell number) for its translation to the clinical setting. The discovery of a specific set of striatal PV+ neurons, organized as a functional ensemble, responsible for production of most of the striatal GDNF, offers a well-identified target to stimulate endogenous production of GDNF. This electrically (gap-junction) interconnected PV+ neuronal pool is particularly attractive, as stimulation of a few of these cells could induce a synchronized activation of the whole population. However, the actual role of PV+ cells in nigrostriatal protection and the functional relations between the different subclasses of interneurons (GABAergic and cholinergic) need to be evaluated by selective deletion of the *Gdnf* gene in each one of these cell types. In addition to the striatum, PV+ neurons are also present in other parts of the brain, in particular in the cerebral cortex. As cortical PV+ neurons do not significantly produce GDNF, it would be interesting to investigate molecular differences between cortical and striatal PV+ neurons that make the latter capable of producing GDNF. Most of the research on GDNF has been done on non-human samples and models. The actual role of human striatal GDNF and the identification of human striatal cells producing this trophic factor are questions that should be urgently addressed by experimental work. GDNF therapy holds much hope and still remains an important field of investigation in PD. Combined with early diagnosis, neuroprotection by endogenous GDNF stimulation may be a potential preventive therapy to PD patients.

## Conflict of interest statement

The authors declare that the research was conducted in the absence of any commercial or financial relationships that could be construed as a potential conflict of interest.
